# The complete chloroplast genome of *Semiaquilegia guangxiensis*, a rare and endemic herb in Guangxi, China

**DOI:** 10.1080/23802359.2020.1773336

**Published:** 2020-06-05

**Authors:** Xin-Mei Qin, Zhang-Ping Huang, Yong-Bin Lu, Qiang Zhang

**Affiliations:** Guangxi Key Laboratory of Plant Conservation and Restoration Ecology in Karst Terrain, Guangxi Institute of Botany, Guangxi Zhuang Autonomous Region and Chinese Academy of Sciences, Guilin, China

**Keywords:** Chloroplast genome, *Semiaquilegia guangxiensis*, Trib. Isopyreae, phylogenetic analysis

## Abstract

The complete chloroplast genome sequence of *Semiaquilegia guangxiensis* was assembled and the phylogenetic relationship with other species in Trib. Isopyreae was inferred in this study. The chloroplast genome is 164,047 bp in length. A typical quadripartite structure was detected, including two inverted repeats (IRs) of 29,581 bp, which are separated by a large single-copy (LSC) and a small single-copy (SSC) of 87,490 bp and 17,395 bp, respectively. Moreover, The genome comprises 132 genes, including 88 protein-coding genes, 36 tRNA genes, and 8 rRNA genes. The overall GC content of the chloroplast genome is 38.9%. The phylogenetic analysis indicated that *S*. *guangxiensis* is most closely related to its congener *S*. *adoxoides*, and *Semiaquilegia* is most closely related to *Aquilegia* in Ranunculaceae.

*Semiaquilegia* Makino was assumed to consist of a sole species in the family Ranunculaceae till to the recent past, namely *S*. *adoxoides* (DC.) Makino – a perennial medicinal herb with tuberous root. It is distributed in Asian subtropic and temperate regions in China, Korea, and Japan, growing in various habitats of fields, roadsides, valleys, slopes, etc. (Fu and Orbélia 2001). In recent years, three new species of *Semiaquilegia*, namely *S*. *guangxiensis* Yan Liu & Y.S. Huang, *S*. *quelpaertensis* D.C. Son & K. Lee, and *S*. *danxiashanensis* L. Wu, J.J. Zhou, Q. Zhang & W.S. Deng have been described (Huang et al. 2017; Son et al. 2017; Zhou et al. 2019). Among them, *S*. *guangxiensis* is endemic to broad-leaved forests of limestone hills in Guangxi, China; *S*. *quelpaertensis* grows in submontane broad-leaved forests in Korea; and *S*. *danxiashanensis* is restricted to Danxia landform in Guangdong, China. However, the complete chloroplast genomes have not been reported in this genus yet except for *S. adoxoides* (Zhai et al. 2019). Here, we report the complete chloroplast genome of *S*. *guangxiensis*, and the inferred phylogenetic relationships of this species as well as the genus *Semiaquilegia* to other taxa in Trib. Isopyreae.

Total DNA was extracted from the silica-dried leaves of *S*. *guangxiensis*, which was collected from Yongfu county, Guangxi, China (24°56′10″N, 110°6′46″E). The voucher specimen was deposited at the Herbarium of Guangxi Institute of Botany (IBK00421269). The genomic paired ends (PE150) sequencing was performed on NovaSeq 6000 (in Novogene corp., Tianjin, China). Approximately 2.4 Gb of clean data were obtained. *De novo* assembly were employed with SPAdes 3.11.0 (Bankevich et al. 2012). Annotation was performed using PGA (Qu et al. 2019) with reference to *S*. *adoxoides* (MK569498).

The chloroplast genome of *S*. *guangxiensis* (GenBank accession number: MT410682) has a total length of 164,047 bp with an overall GC content of 38.9%. The cp genome contains one LSC region of 87,490 bp and one SSC region of 17,395 bp, which are separated by two IR regions of 29,581 bp, respectively. A total of 132 genes are encoded for the chloroplast genome, which include 88 protein-coding genes, 8 rRNA genes, and 36 tRNA genes. In these genes, six tRNA genes (*trnK*-*UUU*, *trnG*-*UCC*, *trnL*-*UAA*, *trnV*-*UAC*, *trnI*-*GAU*, *trnA*-*UGC*) and nine protein-coding genes (*atpF*, *ndhA*, *ndhB*, *petB*, *petD*, *rps16*, *rpl16*, *rpl2*, and *rpoC1*) contain one intron, each of the two gene (*clpP*, *ycf3*) has two introns, and *rps12* gene has trans-splicing.

To understand the phylogenetic position of *S*. *guangxiensis* as well as the genus *Semiaquilegia* within Trib. Isopyreae in the family Ranunculaceae, a maximum likelihood tree was constructed by RAxML (Stamatakis 2014) based on the concatenated data of 74 protein-coding genes from the chloroplast genome sequences of 21 species with *Trollius chinensis* and *T*. *ranunculoides* selected as outgroups according to the larger-scale phylogenetic studies in Ranunculaceae (Zhai et al. 2019). The phylogenetic tree shows that *S*. *guangxiensis* is most closely related to *S*. *adoxoides* (BS = 100%), and *Semiaquilegia* is most closely related to *Aquilegia* in Ranunculaceae (BS = 100%) (Figure 1).

**Figure 1.**
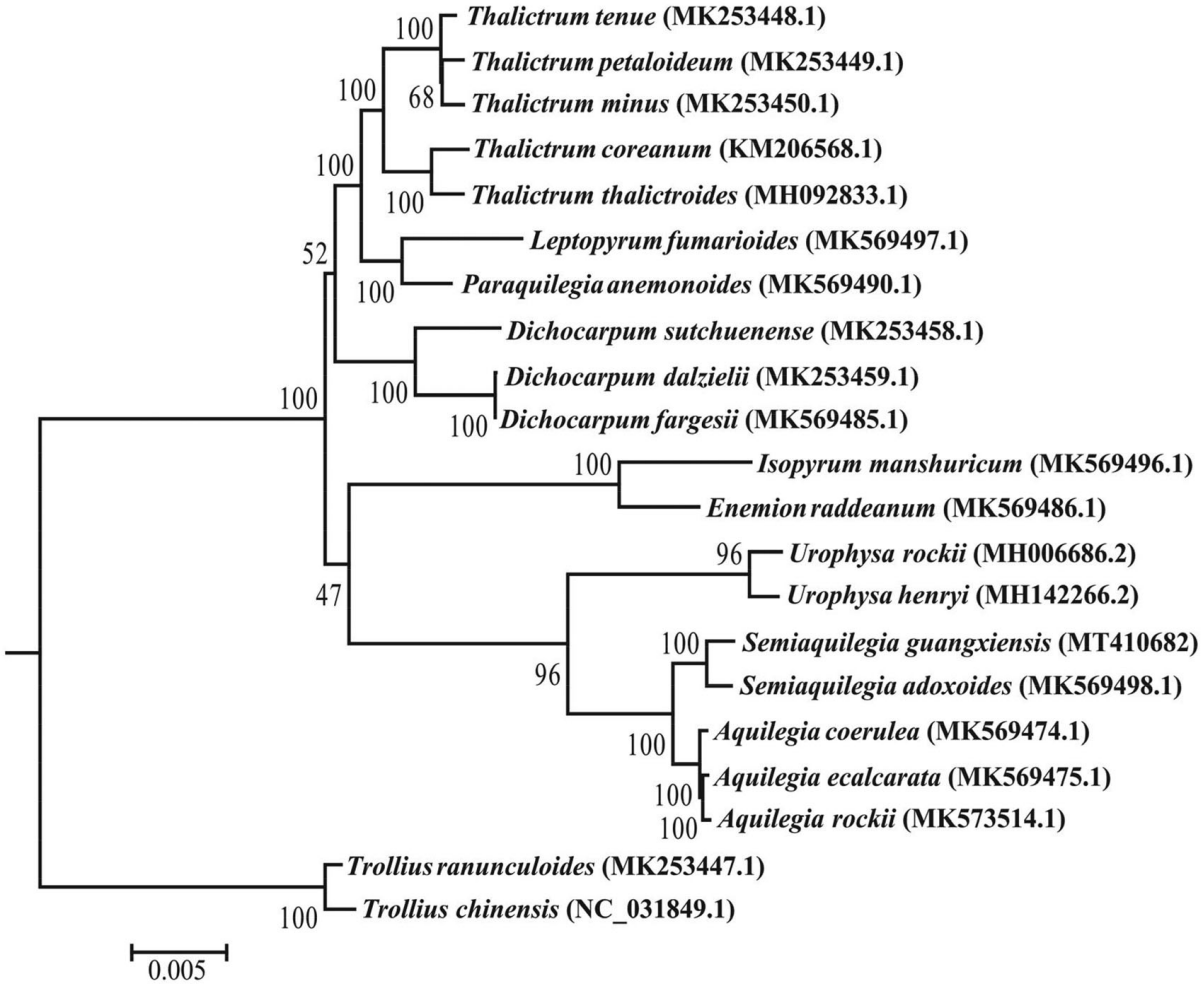
ML Phylogenetic tree of 19 species of Trib. Isopyreae reconstructed based on the concatenated data of 74 protein-coding genes. Bootstrap support values (10,000 replicates) are shown at the nodes.

## Data Availability

The complete chloroplast genome sequence of *S. guangxiensis* has been submitted to the GenBank (https://www.ncbi.nlm.nih.gov/genbank/), and the accession number is MT410682. This sequence will be released immediately after process by the NCBI staff. Then, the data that support the findings of this study is openly available in GenBank.
